# A Volunteer Passion: A Qualitative Look at How We Measure and Reward the Work of Medical Educators

**DOI:** 10.7759/cureus.65849

**Published:** 2024-07-31

**Authors:** Cynthia J Stein, Donna Luff, Jessica M Gold, Richard M Schwartzstein, Jennifer C Kesselheim

**Affiliations:** 1 Division of Sports Medicine, Department of Orthopedics, Boston Children's Hospital, Boston, USA; 2 Department of Orthopedics, Harvard Medical School, Boston, USA; 3 Department of Immersive Design Systems, Boston Children's Hospital, Boston, USA; 4 Department of Anesthesia, Harvard Medical School, Boston, USA; 5 Division of Pediatric Hospital Medicine, Department of Pediatrics, Stanford University School of Medicine, Stanford, USA; 6 Division of Pulmonary, Critical Care, and Sleep Medicine, Beth Israel Deaconess Medical Center, Boston, USA; 7 Department of Medicine, Harvard Medical School, Boston, USA; 8 Department of Pediatrics, Dana-Farber/Boston Children’s Cancer and Blood Disorders Center, Boston Children's Hospital, Boston, USA; 9 Department of Pediatrics, Harvard Medical School, Boston, USA

**Keywords:** passion, teaching metrics, work dissatisfaction, career satisfaction, teacher education, teaching, physician satisfaction, academic promotion, medical educators

## Abstract

Background

Medical educators face many challenges, including the absence of defined roles, lack of standard career paths, and limited support in systems that generally prioritize research and clinical productivity over educational activities. Providers also teach to widely varying degrees. This study was designed to specifically examine the professional rewards and obstacles experienced by physicians who have dedicated significant energy and career focus to medical education.

Methodology

A phenomenological approach was used in this qualitative study. Purposeful sampling was utilized to identify medical educators from different institutions and geographical areas. Participants were categorized by gender and career stage. Semi-structured interviews were conducted, and reflexive thematic analysis was used to develop themes across items and participants.

Results

Twenty-two medical educators were interviewed (11 males, 11 females), with an average age of 51 (range: 38-72) years. The average time from completion of training was 18 years (range: <1 to 41 years). Two main themes were constructed, which related to medical educators’ career motivations and challenges: (1) Joy and purpose (subthemes: Interaction with learners, Impact, and Innovation) and (2) Everyone teaches (subthemes: Lack of recognition, Lack of reward, Malalignment of metrics)

Conclusions

The greatest source of motivation and satisfaction for medical educators is linked to the work itself; in addition to interactions with learners, educators derive pleasure from the innovation, collaboration, and systems thinking involved in their work. Importantly, participants also experience dissatisfaction, primarily due to a lack of recognition and reward, and metrics that do not consistently demonstrate their achievements. Participants provided examples of metrics that more accurately reflected the work of education; they identified clear benefits of academic promotion; and they highlighted significant challenges in the promotional system. The implementation of appropriate systems of measurement and reward is needed to better support the work of medical educators. Our aim should be not only to increase opportunities for satisfaction but also to reduce factors that cause frustration and limit advancement.

## Introduction

As the world of medicine continues to evolve, the roles of healthcare providers, expectations of patients and communities, and requirements of trainees must also change. Medical educators are uniquely positioned to adapt, guide, and transform the educational system to help meet these expanding needs. Despite calls for more medical educators [1] and predictions of growing medical educator job responsibilities [2], significant impediments persist in the methods by which medical schools and academic medical centers recognize, promote, and support medical educators [3]. 

Medical educators as a group face many challenges. There is no clear definition or qualification of a *medical educator*. There is no standard entry route into the field, structured career path, or clearly defined roles [4], and medical education may be viewed by some as less prestigious than other career tracks [5]. Medical school faculty have described the challenges of competing demands and lack of protected time for educational work [6]. Institutions often recognize and reward research productivity and clinical work over contributions to medical education [4,7]. Therefore, notable financial disincentives often exist for involvement in teaching activities [8]. 

Especially given these challenges, work has been done to better understand why physicians teach or choose not to [4,9-17]. However, the majority of the literature has focused either on community physicians or on researchers and clinicians at academic medical centers who are involved in teaching to varying degrees, rather than on the experiences of physicians who have made medical education a major career focus. A recent study identified sources of joy specifically in medical educators in terms of factors that allow them to flourish [18].

The goal of this research was to learn how academic physicians dedicated to medical education view their careers and to identify factors that can support and incentivize medical educators, looking both at positive influences that lead to work satisfaction and negative factors, which cause dissatisfaction.

Theoretical framework

Using Herzberg’s Motivation-Hygiene theory as a starting point, this qualitative study explores medical educators’ perceptions of their work experiences and career trajectories. Herzberg theorized that rather than being on opposite ends of a spectrum, workplace satisfaction, and dissatisfaction are affected by separate factors rooted in different human needs.

Satisfaction factors, termed motivators, are tied to the need to grow and develop. They are inherent to the work itself and sources of job satisfaction: “achievement, recognition for achievement, the work itself, responsibility, and growth or advancement” [19].

Dissatisfaction factors, termed hygiene elements, relate to the need to avoid pain and discomfort, and are often linked to the context in which work is done: “company policy and administration, supervision, interpersonal relationships, working conditions, salary, status, and security” [19].

This theory suggests that people can be satisfied with certain parts of their work and discontented with other parts. The degree to which individuals are impacted by specific factors, and the extent to which certain factors outweigh others, can vary from person to person.

## Materials and methods

Study design

The study protocol was submitted to the university's Internal Review Board and was determined to be exempt. A phenomenological approach was chosen for this qualitative study to examine participants’ understanding of their experiences as medical educators [20]. A semi-structured interview guide was developed around the concepts of Herzog's theory of work motivators and obstacles. The guide was pilot-tested for clarity, significance, and relevance of the questions. The semi-structured format allowed for follow-up questions to enhance understanding of participants' perceptions and experiences. 

Recruitment

Medical educators in the United States and Canada with either a DO or MD degree were eligible for participation.

Inclusion Criteria

Inclusion criteria included one or more of the following: (1) having a paid role as a medical educator or receiving salary support for educational work, (2) holding an advanced degree in education, medical education, or health professions education, or (3) having a publication in the area of medical education. 

Exclusion Criteria

Participants were excluded from the study if they met any of the following criteria: (1) they were a non-physician medical educator, (2) they were unable to complete the interview within the study period, or (3) they lacked fluency in English.

Purposeful sampling was used to recruit potential participants identified through personal and professional contacts and professional websites, categorized by gender and career length. Potential participants were contacted by email and invited to participate. Recruitment was done on a rolling basis. 

Data collection

Demographic data were collected via an online survey distributed at the time of interview scheduling. Interviews were conducted by a single interviewer (CJS) between October 2022 and March 2023 via video calls. The interview guide was used to conduct semi-structured interviews and allowed for probing for additional information and follow-up on interesting points during the interview. With participant permission, the interviews were recorded, transcribed verbatim, and anonymized. Participant numbers were used to maintain anonymity. 

Analysis

Inductive reflexive thematic analysis was used to identify patterns in the data, using the six stages described by Braun and Clark [21,22] to describe patterns and identify broader applications of the data. This iterative process allowed for multiple readings of the transcripts. Themes and subthemes were compared within and between participants. Quotations were selected to illustrate and support this categorization into themes and subthemes. 

Standards for Reporting Qualitative Research (SRQR) [23] were used to guide the work and improve the reporting of findings.

## Results

Thirty-two medical educators were invited by email to participate: six did not respond, one did not meet inclusion criteria, and three indicated interest but did not schedule an interview within the study period. Twenty-two (68.8%) people agreed to participate, were interviewed, and were included in the analysis. At the point at which recurrent themes were becoming evident through analysis, a total of 22 interviews had been scheduled. The decision was made to complete as many of the scheduled interviews as possible within the 6 month study period. Interviews ranged from 26-75 minutes in length. 

Eleven participants identified as female and 11 identified as male. See the distribution of participants by gender and career stage in Table 1, and refer to participants' demographic details in Table 2. Eight participants (36%, 4 females, 4 males) identified as belonging to groups underrepresented in medicine (URM). The average age at the time of interview was 51 (range: 38-72) years. All participants held MD degrees. One had both an MD degree and a PhD degree. Participants were trained in 18 different general, specialty, and subspecialty fields. They came from 16 hospitals and 12 US states. 

**Table 1:** Distribution of participants by gender and career stage

	Early Career (<10 years)	Mid Career (10-25 years)	Advanced Career (>25 years)	Total
Female	2	8	1	11
Male	3	4	4	11
Total	5	12	5	22

**Table 2:** Participant demographic information. Early: early career <10 years, Mid: mid-career 10-25 years, Adv: advanced career >25 years 1: professor, 2: associate professor, 3: assistant professor, 4: instructor, 5: adjunct G, generalist; S, specialist/subspecialist; IM, medicine; Peds, pediatrics; Rad, radiology; Gen Surg, general surgery; EM, Emergency Medicine; URM, underrepresented in medicine

Participant number	Gender	Age (years)	Primary field	URM	Career stage	Academic rank	Institution location
01	M	42	G: IM	N	Mid	2	NC
02	F	51	S: IM	N	Mid	1	NC
03	M	39	S: Peds	Y	Early	4	MA
04	F	63	S: IM	N	Adv	1	MI
05	M	72	S: Peds	N	Adv	1	CO
06	F	46	S: Rad	N	Mid	1	WI
07	F	53	S: Peds	N	Mid	1	OH
08	F	39	S: Peds	Y	Early	4	MA
09	M	46	S: Gen Surg	N	Mid	2	MA
10	M	49	G: Peds	N	Mid	3	MN
11	F	60	S: IM	N	Mid	2	MA
12	F	38	S: Peds	Y	Early	3	PA
13	M	63	S: Peds	N	Adv	1	UT
14	M	61	S: IM	N	Adv	1	NV
15	F	47	S: Peds	N	Mid	3	MA
16	M	55	S: IM	N	Mid	2	IN
17	M	63	S: Peds	Y	Adv	1	OH
18	M	31	S: EM	Y	Early	5	MI
19	F	53	S: Peds	Y	Mid	3	MA
20	F	52	G: IM	Y	Mid	2	MA
21	F	53	S: IM	N	Mid	1	NC
22	M	38	G: IM	Y	Early	2	OK

Using Herzberg’s motivational hygiene theory as a foundation, two overarching themes, each with three subthemes, were constructed based on patterns that transcended interview questions and were noted across participants (Table 3)

**Table 3:** Satisfaction and dissatisfaction factors.

Satisfaction/motivation factors: Joy and purpose	Dissatisfaction/hygiene factors: Everyone teaches
Interaction with learners	Lack of recognition
Impact on the system	Lack of reward
Innovation	Malalignment of metrics

Joy and purpose

Medical educators expressed gratification from different facets of their work, which formed the basis of their self-evaluations, how they measure their achievements, and the goals they set for their work. A few participants described an internal drive to teach and to see the world through the framework of education. This enjoyment centered on three subthemes. The subtheme referred to by almost all participants was Interaction with Learners. They described the pleasure of working directly with trainees and being actively involved in the process of learning and development. They felt the satisfaction of being able to observe the evolution of the learner, sometimes over short periods, sometimes over many years. They also enjoyed the benefits of advancing their knowledge alongside their learners.

In addition, respondents reported the larger, system-based Impact of their work. They reflected on their ability to influence the educational system, to contribute to the development of future generations of doctors, and to increase their contributions indirectly through the work of their students. Included in this subtheme, and described in detail by several participants, was a desire to create system-based change and improve the learning environment.

Some participants highlighted the opportunities for Innovation and investigation in their work: formulating interesting questions, designing studies and interventions, and creating new methods and tools. They commented on their enjoyment of working with colleagues in medical education, and multiple participants included the importance of publication for dissemination of research and to advance the field of medical education. Illustrative quotes are given in Table 4.

**Table 4:** Illustrative quotes related to work satisfaction/motivation factors.

Joy and purpose	
Interaction with learners	“…the one constant over the years was teaching the residents, and really, that’s why I’m here. I mean that’s the one thing that I really enjoy doing….” (p. 5)
	“…seeing their growth is probably the most exciting part of it, […] a sense of everything's new to them. I think that's exciting, just to see things through their eyes.” (p. 18)
	“I liked being around learners and I liked co-producing knowledge with them. So when I say I like to teach, I don’t mean so much ‘I like to stand up in front of a group of people and lecture to them.’ I just like to be around learners, considering myself a lifelong learner.” (p. 20)
Impact	“I think of it as like a cascade, so I influence 10 people, and then they may influence two or three more people and then those two or three people may influence more and so in the end, it's just like fireworks, just a bunch of bright lights of medical education all over the country.” (p. 21)
	“My biggest goal is to have a great learning environment and a great teaching environment. So for learners, I just want them to feel like they are being accepted for who they are and helped to develop into the career that they would like to have […] and I would love it if my program directors had appropriate support to be able to feel like they could innovate and really do great stuff in education.” (p. 2)
	“As an educator, you […] get credit for what all of your students do […] And I think that’s the coolest thing to be able to say, ‘I helped make this person an amazing educator’ or ‘I helped make this person an amazing physician.’” (p. 1)
Innovation	“…education is not only teaching, it’s also being creative. It's also being a designer. It's also being a change in our patients' outcome.” (p. 17)
	“I like working with really, really smart, talented people, who ask good questions. They push the envelope. They ask, ‘I know this is the way it's always been done, but can we do it a little differently?’ And I think, realizing also that the way it's been done is not always the best way that has been done, and so trying to […] rethink about problems. There's always problem solving, which I love.” (p. 15)
	“I enjoy working with other educators. Those times where we’re in the think tank and we’re like ‘Let’s think about this different curriculum and what we’re trying to do.’ Those are my favorite times as well.” (p. 12)

Everyone teaches

Many participants in this study also reported persistent challenges and causes of dissatisfaction. They discussed the expectation that everyone in academic medicine is involved in teaching, and the perception of some that the work of medical educators is not unique. Their responses provided examples of how they felt medical educator contributions were minimized or underrecognized. These were categorized into three subthemes.

The first was a Lack of recognition of the role of a medical educator. One participant described aspects of their work in medical education as “invisible.” Many participants felt that part of the distinction between a medical educator and others who teach is a person’s self-identification and work focus, especially in the areas of educational scholarship, consideration of the educational system, and use of learning theory and methods.

Relatedly, some participants indicated a Lack of reward. Not only did many feel that their time and effort were not well compensated, some recognized a clear cost to focusing time and energy on medical education. They described a lack of dedicated, protected time for medical education work, a lack of financial and administrative support, and obstacles to promotion. One participant referred to medical education as "a volunteer passion.”

About a third of participants (eight of 22) felt that they were adequately recognized and rewarded for their medical education work. Of these, six were full professions, five females/three males, and two identified as underrepresented in medicine.

A few participants brought up inequalities in pay between specialties, and they noted that, when compensated, teaching is often paid at a rate lower than that for clinical work.

Also affecting the existing reward systems, a few participants commented on the underlying conflicts in the priorities of the academic medical center’s mission and the fact that the educational goals may not be top priority. Mentioned specifically by several participants was the perception that institutions saw education as a cost, “something they are forced to pay for.”

From here emerged a third subtheme: Malalignment of metrics. Especially in the area of promotion, many participants felt they were being measured primarily in terms of publications and grant funding, without recognition of direct teaching, curriculum development, or program administration. The majority of participants had published in medical education from few to over 100, primarily in traditional print journals, and several had published on-line and/or used social media for their medical education work.

Some participants provided examples and suggestions for better fitting metrics. They also described the efforts that some institutions have made to improve evaluations and the promotional process for medical educators. Examples included educational portfolios and measures of productivity beyond publications, such as Educational Value Units (EVUs), teaching sessions, courses, curricula, presentations, and committee service. Specific participant quotations are given in Table 5.

**Table 5:** Illustrative quotes related to dissatisfaction/hygiene factors.

Everyone teaches	
Lack of recognition	“From a reputational standpoint especially at big institutions, you're not the major researcher, you're not the major clinician bringing in clinical dollars. You just don't get the respect, if you will, that other groups do. Again, we're medical schools, that's what we are, so that education piece is really very, very important.” (p. 14)
	“I think people feel that education is almost like a pseudoscience in many ways. They don’t see the value of it until they have an education problem…” (p. 9)
	“…depending on the institution, it can be really hard for people to recognize a successful career of a medical educator.” (p. 7)
Lack of reward	“We have fellowship directors who, if they take the 25% to 30% time that they need to be an effective fellowship director and lose that clinical RVU, they actually are losing money to…teach.” (p. 16)
	“…the part that frustrates me the most […] it is just trying to get respect and time from the institution…” (p. 11)
	“…it’s sometimes hard for people to get to the point where you are compensated to spend time doing medical education […] there are a limited number of […] compensated roles out there.” (p. 4)
Malalignment of metrics	“I think they measure my work in number of publications and national presentations and grant funding. In the promotional track, there's not really a ‘Oh you taught 170 hours this year. Great! So we're gonna move you up.’ There's nothing like that.” (p. 8)
	“We are held to unfair standards as educators because […] if you think about promotion as a sort of job assessment, […] one of the things that makes assessment fair is fitness for purpose. And is counting somebody’s publications, is that fit for purpose?” (p. 20)
	“…what in medical education are the metrics that we should be using? […] what is more important, impact versus publishing? And I believe in publishing, but I do feel it needs to be a “both-and” somehow…” (p. 3)

Most of the medical educators in this study felt that promotion was important, even *critical*, for a number of reasons, including external validation, credibility, status, income, and eligibility for other positions. They also described the significance of promotion for role modeling and gaining influence necessary to help others. Additional benefits included improving the culture and diversity of academic medicine. Different systems of promotion were described, and at least one participant indicated that they were promoted on a clinical tract, rather than an education tract. A few commented that, in practical terms, promotion would not change their day-to-day work. Specific quotes related to promotion are given in Table 6.

**Table 6:** Illustrative quotes related to the significance of academic promotion.

Significance of promotion
“…some of the reason I wanted to push for being a full professor was because, as you know, in order to get promoted, you have to get letters from people who are a higher academic rank than you.[…] So to me, some of it was that external validation of recognizing that I'm valuable as an educator, but some of it was being able to help my colleagues around the country. Because if clinician educators aren't promoted, then clinician educators won't get promoted.” (p. 2)
“We also do not represent the population of patients that we serve, and so I think getting people promoted in those areas is incredibly important. Demonstrating, first of all that we value diversity, but then also, I think it helps both our learners […] being able to see themselves in the faculty that will be teaching them, and not just at lower levels, but in leadership positions […] then, for the patients, we take care of as well.” (p. 16)
“I think that your status - so whether you’re an instructor or an assistant, or associate, a professor - I think to some level that is a statement about your accomplishments and success in this academic institution. So, I get that. I think from an actual practical standpoint, it’s not so important. It’s not going to see the patient. It’s not going to be sitting there with me […] when I’m making determinations about things…(p. 19)

Subgroup analysis showed that, other than the relationship between feeling one’s work is recognized and the academic rank of professor described above, no patterns of participant gender, race, or academic rank could be found that corresponded to the various responses.

## Discussion

This qualitative study explores the motivations and challenges of 22 physician medical educators. It reinforces what we know about satisfying aspects of medical education and introduces additional creative elements of the work not well described previously. It also demonstrates that, despite a growing structure and focus on medical education, many study participants still perceive a lack of adequate acknowledgment, support, and compensation. Not previously reported, study participants described the multiple benefits of promotion. In addition, the publication discussed research, dissemination, and contributions to the field of medical education, rather than as an accurate metric of educational impact.

For the participants in this study, the answer to the question “What motivates medical educators?” is the work itself. They find their work to be interesting, meaningful, and challenging. They describe a personal sense of achievement and opportunities for growth. Enjoyment of the process of teaching, repaying one’s teachers, and gratification in training future generations of physicians have been well documented in the literature [13,15,16]. Additional reasons such as intellectual stimulation [24] and intellectual satisfaction [10] have also been reported, and Lagina et al categorized many of these factors [18] within the PERMA model (Positive Emotion, Engagement, Relationships, Meaning, and Accomplishment) defined by Seligman [25]. The medical educators in this study reinforce the importance of many of these same factors and also highlight the creativity, innovation, and investigation that are vital parts of medical education.

As Herzberg postulated, one can experience both great satisfaction and significant dissatisfaction. In this study, dissatisfaction centered on a lack of protected time and limited funding for medical education work. As a result of - and likely also contributing to - this continued lack of institutional recognition are inadequate metrics to quantify the accomplishments and impact of their work. These findings of continued challenge are particularly notable because they persist despite tremendous changes in the training and organization of medical educators over the past few decades.

About two-thirds of participants (14/22) provided comments that related to a lack of recognition, an undervaluation of their work, or insufficient protected time. These numbers are not presented to suggest that this finding is generalizable to all medical educators, but rather to show that these sentiments were commonly expressed by participants of this study. They are also consistent with the findings that some medical educators report feeling “undervalued” and “poorly compensated” [18]. To better understand why some perceived this lack of respect when others did not, the discrepant cases were examined. Of the 8 participants who indicated that their work was well recognized and rewarded, there was a marked association with academic rank with six of the eight being full professors. It is possible that those who work in systems where their efforts are best recognized also receive academic promotion as part of that recognition. It is also possible that with promotion, these medical educators felt they received the recognition they deserved. As examples of support, some participants described mentorship, training programs, and compensated time for medical education. They illustrated how newer metrics have been developed to encompass the work of medical educators, such as educator portfolios, educational relative value units (eRVUs), medical educator award programs, and promotional tracks for educators. These data suggest that some changes are occurring. These types of measurements that focus on the products of educational work could be expanded. It would be interesting for future research to examine how different promotional and incentive models affect the productivity, motivation, and satisfaction of medical educators, especially across different medical systems in different areas of the world.

Whether or not academic promotion is a motivating factor for medical educators has not previously been reported to the best of our knowledge. It has likely been assumed that, as part of the academic community, medical educators would seek promotion. The data from this study demonstrate that participants did feel academic promotion was important. They were acutely aware of the benefits of promotion, such as elevated status, increased salary, acknowledgment of achievements, eligibility for higher level positions, ability to support the promotion of others, the chance to act as a role model, as well as the opportunity to increase diversity and improve the culture in academic medicine. From the participants' descriptions, it was also clear that the pathways to promotion and criteria for academic advancement vary from institution to institution. 

Given the known gender and racial disparities in academic medicine [26-28], it was anticipated that there would be notable differences in this study between the experiences of women and men, and between groups under-represented and well- or over-represented in medicine. However, among the participants in this study, differences were not detected by gender or race around the specific topics of gratification and challenges in medical education. In some cases, a given point of view was so widely shared, such as the importance of promotion, that there were no differences to dissect. In other cases, like the question of recognition and protected time for medical educators, where participants did have different experiences, no patterns were found by gender, race, or career stage, to correlate with these different viewpoints.

Eghosa-Aimufua et al. [29], in a study of both clinical and nonclinical women in medical education, described positive influences, such as the *impact of mentorship* and *importance of meaningful networks*, which were also noted by participants in this study. However, the inflection points that, for some women in the earlier study, led to a greater focus on medical education - pressure to work full time, personal crisis, prior negative experiences - were not described by participants in this study, female or male. Participants in this study described working toward a goal of medical education or a gradual shift in focus, rather than a more drastic or forced change. The differences in findings between the studies may be due to the focus on career transitions in the earlier research (vs. satisfaction and obstacles in this study); inherent differences in the participants; or the differences in work requirements and expectations in different systems.

Limitations

The inclusion criteria for this study necessarily limited the participants to certain backgrounds, and therefore, other types of medical educators were not included, the most obvious being nonphysician medical educators. Similarly, interviews were conducted in English and therefore medical educators who were not fluent in English would not have been eligible for participation. 

The participants were selected based on gender and career stage. The largest group was comprised of full professors, possibly overrepresenting their perspectives. However, there were many similarities in the experiences of participants across career stages. Other than the noted association of feeling recognized and rewarded with the rank of full professor, no other patterns were found that mapped specific findings to gender or career stage.

It is possible that there were no differences between participants in the specific topics explored in this study; that the interview questions did not effectively elicit existing differences; or that differences were not apparent due to the effects of other factors, such as career stage, since the majority of participants who self-identified as belonging to underrepresented groups in medicine were also in the early career stage (five early, two mid, and one advanced).

It is also possible that geographic bias could have had an impact on study results. Participants worked in 12 different states across the country; however, the largest group came from the Northeast United States, and seven participants were in a single state.

## Conclusions

Medical educators in this study described many factors that motivate and inspire their work. In addition, this study reveals that significant career challenges remain, especially in the areas of recognition and evaluation of the work of medical educators. The importance of academic promotion was highlighted in terms of acknowledgment, role modeling, access to higher level positions, and the ability to assist others. As identified by study participants, improvements in the current systems of support, assessment, and reward have great potential to reduce career obstacles for medical educators.
